# Selection Bias in Digital Conversations on Depression Before and During COVID-19

**DOI:** 10.2196/42545

**Published:** 2023-11-20

**Authors:** Edward Lee, Davin Agustines, Benjamin K P Woo

**Affiliations:** 1 College of Osteopathic Medicine of the Pacific Western University of Health Sciences Montrose, CA United States; 2 Olive View–University of California, Los Angeles Medical Center Sylmar, CA United States

**Keywords:** depression, COVID-19, treatment, race, ethnicity, digital conversations, health belief model, artificial intelligence, AI, natural language processing, NLP

## Letter to the Editor

We read with great interest the article, “Applying the Health Belief Model to Characterize Racial/Ethnic Differences in Digital Conversations Related to Depression Pre- and Mid-COVID-19: Descriptive Analysis” by Castilla-Puentes et al [1]. The authors’ aim was to use digital conversations obtained by CulturIntel to describe the mentality, key drivers, and obstacles related to depression before and during the COVID-19 pandemic mapped to health belief model (HBM) concepts. The article concluded that there were substantial racial and ethnic differences that facilitated or hindered seeking help and treatment for depression before and during COVID-19. We applaud the monumental task of evaluating a large number of data points and categorizing them according to population. However, even though the authors acknowledge the limitation of having used only digital conversations, we wish to address two distinct issues related to demographics: (1) difficulties in addressing older populations’ needs and (2) identification of racial or cultural groups.

First, the sole use of digital conversations results in a selection bias against older adults. According to a Pew Research Center report from 2021, only 45% of those aged ≥65 years used social media sites compared to 84% of those aged 18 to 29 years and 81% of those aged 30 to 49 years [2]. Other methods should be implemented to capture older adults’ health beliefs and conversations about depression in order to obtain a more accurate representation of the population to create an HBM. Furthermore, the 2019 US Census Bureau reported that there were 54.1 million residents aged ≥65 years in the United States [3]. These points highlight the need for diverse methods to numerically capture this important segment of society.

Second, despite categorizing data according to race and ethnicity, residency status was not identified. There is a difference between US-born, naturalized, and noncitizen immigrants. Asian immigrants who are in the process of applying for citizenship or are ineligible for citizenship are considerably more depressed than naturalized citizens due to the fear of possible deportation [4]. The rapid growth of various Asian subgroups, each with different cultural acclimatization needs, highlights the importance of cultural awareness in addressing mental health needs. Further categorization of Asian subgroups revealed that both noncitizens and naturalized citizens reported worse overall mental health, as well as health in general, compared to their US-born counterparts. The differences are attributable to multiple socioeconomic factors including education level, employment, insurance, and access to health care [5]. Unfortunately, the use of natural language processing to categorize racial/ethnic groups cannot discern differences across cultural generations. Methods that can capture specific Asian languages and ways to identify residency status should be developed and used to help identify generational and cultural differences. Implementing methods to cover the points mentioned can strengthen future applications of HBM studies.

In conclusion, we believe that addressing the above concerns will create an enhanced, culturally competent HBM that is capable of identifying specific populations as well as needs for mental health support. We look forward to future advancements in this field.

## Abbreviations

HBM: health belief model
